# *Brassica rapa* L. (Turnip) Ethanol Extract Alleviates Hyperuricemia by Modulating Purine Metabolism Enzymes and Renal Urate Transporters

**DOI:** 10.3390/foods15173108

**Published:** 2026-09-01

**Authors:** Yan Wang, Yuheng Yang, Yinghao Liu, Dongyuan Cheng, Wenwu Yao, Abdulla Yusuf, Kai Yang

**Affiliations:** 1 College of Food Science and Technology, Zhejiang University of Technology, Hangzhou 310014, China; wangyan062006@zjut.edu.cn (Y.W.); yangyuheng1196@gmail.com (Y.Y.);; 2 Zhejiang Provincial Center for Disease Control and Prevention, Hangzhou 310051, China; 3 Laboratory of Xinjiang Native Medicinal and Edible Plant Resource Chemistry, College of Chemistry and Environmental Science, Kashi University, Kashi 844000, China

**Keywords:** *Brassica rapa* L. ethanol extract, hyperuricemia, xanthine oxidase, urate transporters, molecular docking

## Abstract

Hyperuricemia (HUA) is a metabolic disorder caused by excessive uric acid (UA) production and/or insufficient UA excretion. *Brassica rapa* L. (turnip) is a traditional medicinal and edible plant in northwestern China. In this study, the urate-lowering effects of *B. rapa* L. ethanol extract (BrEE) and associated underlying mechanisms were investigated using a hypoxanthine/potassium oxonate-induced HUA mouse model and UA-stimulated HK-2 cells. BrEE was prepared and characterized; quercetin-3-glucuronide was the most abundant compound in BrEE, with a relative abundance of 22.04%. BrEE significantly lowered serum UA levels and improved renal function in mice. Key UA-producing enzymes, xanthine oxidase (XOD) and adenosine deaminase (ADA), were suppressed and renal UA transporters were modulated to enhance excretion: URAT1 and GLUT9 were downregulated and ABCG2 was upregulated. These results indicated that BrEE’s protective effects were associated with its regulatory effects on UA transport-related protein expression. BrEE also alleviated oxidative stress, and reduced inflammation and renal pathology. Meanwhile, molecular docking predicted favorable binding between the major BrEE constituents and XOD or URAT1, providing preliminary support for their potential interactions with these targets. Thus, BrEE exerted multi-target urate-lowering and kidney-protective effects, providing preliminary evidence for the potential of *B. rapa* L. as a plant-derived resource for further investigation in the development of functional food ingredients to regulate urate metabolism.

## 1. Introduction

Hyperuricemia (HUA) is a common metabolic disorder characterized by the abnormally elevated level of serum uric acid, which is primarily caused by excessive uric acid production or impaired uric acid excretion from the human body [1]. Recent shifts in the human dietary structure have caused increased purine uptake, resulting in a marked increase in the global prevalence of hyperuricemia [2]. Thus, HUA is a critical metabolic challenge, closely trailing type 2 diabetes in terms of its public health impact [3]. In China, the overall HUA prevalence among adults has reached 17.7% and continues to increase annually [4]. More importantly, chronic hyperuricemia often culminates in gout, resulting in gouty arthritis, gouty nephropathy, and other complications [5]. HUA also exhibits a complex interplay with chronic kidney disease, hypertension, and various metabolic derangements, including diabetes and metabolic syndrome [6,7,8].

The dynamic balance between UA production and excretion is a crucial physiological mechanism that maintains UA homeostasis in the body. Consequently, disruption of this equilibrium serves as the primary driver for hyperuricemia and its associated pathologies [9]. Uric acid is primarily produced in the liver. Preceding this, adenine-based nucleosides are first deaminated by adenosine deaminase (ADA) [10]. These metabolic intermediates undergo oxidation to form xanthine and subsequently UA. This final stage is governed by xanthine oxidase (XOD), which serves as the rate-limiting enzyme that dictates the total output of UA [11,12]. Renal excretion accounts for roughly two-thirds of the total amount of UA eliminated daily, with excretion of the resulting urine being the primary exit route. The remaining one-third is primarily cleared through the gastrointestinal tract [13]. Urate transporter 1 (URAT1) and glucose transporter 9 (GLUT9) are the cornerstones of renal UA processing, and are predominantly located in renal proximal tubule epithelial cells, which mediate UA reabsorption. Importantly, their overactivity is closely linked to primary HUA pathogenesis [14]. As a major protein responsible for UA discharge, the ATP-binding cassette transporter subfamily G member 2 (ABCG2) is strategically positioned on the apical membranes of both intestinal cells and renal tubules, where it drives the export of uric acid from the body. Substantial UA accumulation often stems from ABCG2 deficiency, whether through functional defects or reduced protein abundance. Such disturbances fundamentally hinder the body’s ability to eliminate uric acid effectively [15].

Allopurinol and benzbromarone serve as the first-line therapies for HUA. Specifically, allopurinol lowers UA levels by suppressing synthesis through XOD inhibition, whereas benzbromarone acts as a uricosuric agent to accelerate renal excretion [16]. Despite their therapeutic potency, the clinical utility of these agents is frequently hampered by significant safety concerns, ranging from cutaneous rashes and gastrointestinal distress to impaired renal function [17,18]. Given the concerns surrounding the safety–efficacy profile of prolonged clinical treatments, the research focus has increasingly shifted toward plant-derived bioactive constituents. These natural compounds represent a viable and potentially safer paradigm for the long-term management of HUA. Different phytochemicals, notably flavonoids, phenolic acids, and polysaccharides, have demonstrated significant uric acid-lowering properties [19,20]. These compounds typically exert therapeutic effects by inhibiting XOD activity, promoting renal UA excretion, or modulating inflammatory pathways [21].

**Figure 1:**
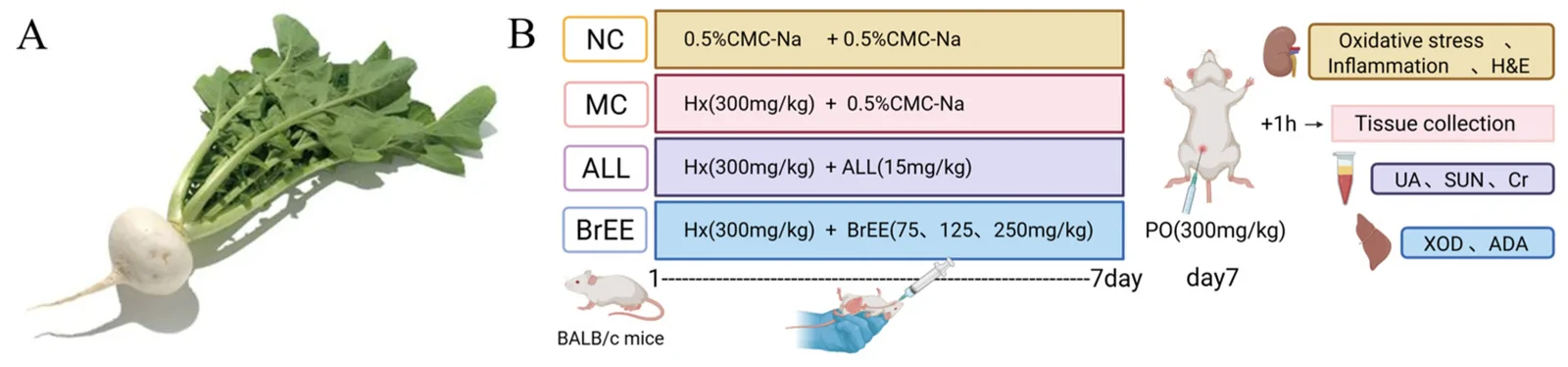
*Brassica rapa* L. (**A**). Schematic showing the experimental design (**B**).

*Brassica rapa* L. (turnip) is a biennial herb of the *Brassica* genus in the Brassicaceae family, and is widely distributed in northwestern China (Figure 1A). In the Xinjiang Uygur Autonomous Region, the herb has a long history of use as both a medicinal and edible plant [22]. Its taproots are commonly consumed directly as everyday vegetables, and can also be processed into pickles, dietary fiber powders, and functional beverages [23]. *B. rapa* L. contains various bioactive components, including flavonoids, polysaccharides, glucosinolates, and vitamins, and exhibits antioxidant, hypoglycemic, anti-asthmatic, and gut microbiota-modulating activities [24,25,26]. However, the effects of *B. rapa* L. ethanol extract (BrEE) in alleviating HUA are unknown, and its therapeutic efficacy and underlying mechanisms remain unclear.

In this study, BrEE was prepared and identified using Ultra-Performance Liquid Chromatography–Quadrupole Time-of-Flight Tandem Mass Spectrometry (UPLC-Q-TOF-MS/MS). A HUA mouse model was also established using hypoxanthine (Hx) combined with potassium oxonate (PO). Serum UA levels, hepatic and renal biochemical parameters, and histopathological changes were evaluated to investigate the potential alleviating effects of BrEE against HUA. Additionally, HK-2 cells were treated with UA to determine BrEE’s protective effects and associated regulatory effects on UA transport-related protein expression. Furthermore, molecular docking indicated that the primary BrEE constituents possess strong binding affinities for both XOD and URAT1, thereby providing molecular evidence supporting its anti-HUA potential. This study provides preliminary insights into the potential of *B. rapa* L. as a plant-derived resource for further investigation in the development of functional food ingredients to regulate urate metabolism.

## 2. Materials and Methods

### 2.1. Chemicals and Reagents

UA, Hx, and PO were purchased from Meryer (Shanghai, China). UA, serum urea nitrogen (SUN), creatinine (Cr), XOD, ADA, malondialdehyde (MDA), superoxide dismutase (SOD), glutathione peroxidase (GSH-Px), catalase (CAT), and reactive oxygen species assay kits and an Annexin V-FITC/PI double staining apoptosis detection kit were purchased from Nanjing Jiancheng Bioengineering Research Institute (Nanjing, China). Allopurinol (ALL) was purchased from Guangzhou Baiyunshan Pharmaceutical Holdings Co., Ltd. (Guangzhou, China). Enzyme-linked immunosorbent assay (ELISA) kits for mouse interleukin (IL)-1β, IL-6, and tumor necrosis factor (TNF)-α were provided by Youke Life Science & Technology Co., Ltd. (Hangzhou, China). Basic DMEM/F12 medium was purchased from Gibco (Grand Island, New York, NY, USA). Fetal bovine serum (FBS) was purchased from Procell Life Science & Technology Co., Ltd. (Wuhan, China). The Cell Counting Kit-8 (CCK-8), 0.25% trypsin–EDTA solution, and penicillin–streptomycin were acquired from Meilunbio (Dalian, China).

### 2.2. BrEE Preparation

Dried *B. rapa* L. powder (Aksu Prefecture, Xinjiang, China) was mixed with 61% ethanol at a ratio of 1:20 (W/V), followed by ultrasonic cell disruption for 24 min. The crude extract was purified using an AB-8 macroporous resin (Macklin Biochemical Co., Ltd., Shanghai, China), washed in reverse osmosis water, and finally desorbed in 70% ethanol (v/v). The resulting solution was concentrated using a rotary evaporator (Shanghai Yarong Biochemical Instrument Factory, Shanghai, China), and the resulting product was freeze-dried to generate BrEE.

### 2.3. BrEE Chemical Constituent Analysis Using UPLC-Q-TOF-MS/MS

BrEE chemical constituents were analyzed using an AB 5600 Triple TOF MS coupled to a UPLC system (Waters H-Class) (Waters Corporation, Milford, MA, USA). A water/acetonitrile gradient (both containing 0.1% formic acid) was employed to resolve BrEE on an HSS T3 column (40 °C) at a flow rate of 400 μL/min. MS/MS data were acquired in information-dependent acquisition (IDA) mode (m/z 50–1200), triggered by precursor ions exceeding 100 counts. For electrospray ionization (ESI) analysis, the source was operated at 550 °C with an ion spray voltage of −4500 V, while the nebulizer and auxiliary gases were maintained at 60 psi [27].

### 2.4. Animal Study Design

We purchased 36 specific-pathogen-free-grade Balb/c male mice (18–22 g) from the Zhejiang Laboratory Animal Center. Ethical approval for the animal procedures in this study was granted by the Zhejiang University of Technology Research Ethics Committee (reference number MGS20241105164). All protocols were strictly conducted in accordance with the Guide for the Care and Use of Laboratory Animals of the Zhejiang University of Technology (Hangzhou, China). After 1 week of acclimatization, the animals were randomly divided into six groups with six mice per group: normal control (NC), model control (MC), ALL, and BrEE low-, medium-, and high-dose groups (BrEE-L, BrEE-M, and BrEE-H, respectively). As shown in Figure 1B, all groups received prophylactic gavage for 7 days prior to modeling. Hx (300 mg/kg) was administered to all groups except the NC group, followed by the assigned treatments 1 h later: ALL (15 mg/kg), BrEE-L (75 mg/kg), BrEE-M (125 mg/kg), and BrEE-H (250 mg/kg), or vehicle control (0.5% sodium carboxymethyl cellulose, CMC-Na). The doses of BrEE were selected based on the effective dose ranges reported for plant-derived extracts in previous experimental studies of hyperuricemia and related renal injury [28]. On day 7, after 2 h of fasting, the mice received an intraperitoneal PO injection (300 mg/kg) to induce HUA, except for the NC mice who received saline. The drugs were administered by gavage to the groups at 1 h after modeling, and then blood and tissue samples were collected at 2 h after gavage [29].

### 2.5. Biochemical Analysis

Serum levels of UA, Cr, and SUN, along with hepatic XOD and ADA activities, were determined using commercial kits according to the manufacturer’s instructions. Renal antioxidant enzyme (SOD, GSH-Px, and CAT) activities and lipid peroxidation (MDA) levels were also measured. Renal IL-1β, IL-6, and TNF-α tissue levels were quantified using ELISA kits.

### 2.6. Histopathology

Following a 24 h fixation in 4% paraformaldehyde, kidney specimens underwent routine histological processing, including dehydration, clearing, and paraffin embedding. After deparaffinization and rehydration, sections were stained with hematoxylin for nuclear staining, differentiated with acid alcohol, and then blued. Sections were then counterstained in eosin for cytoplasmic staining. Stained sections underwent dehydration through graded alcohols and clearing in xylene, before being permanently mounted with neutral balsam. Histological structures on slides were then evaluated using light microscopy [30].

### 2.7. Flow Cytometric Analysis of Apoptosis and Reactive Oxygen Species (ROS)

HK-2 cells are immortalized human proximal tubular epithelial cells that are widely used as an in vitro model to investigate renal tubular injury and related pathophysiological mechanisms. Given that the renal proximal tubule plays an important role in urate reabsorption and transport, HK-2 cells provide a relevant cellular model for investigating the effects of UA exposure and the potential renoprotective effects of BrEE. HK-2 cells (Shanghai Jinyuan Biotechnology Co., Ltd., Shanghai, China) were cultured under standard conditions (37 °C, DMEM/F12 with 10% FBS). Upon reaching the desired confluency, cells were plated at a density of 3 × 10^5^ cells/mL and exposed to 50 μg/mL UA to establish the UA-induced cellular injury model, followed by treatment with BrEE at the indicated concentrations for 24 or 48 h. Potassium oxonate was not added to the culture medium because the in vitro model was designed to investigate the direct effects of UA exposure on renal tubular epithelial cells rather than to reproduce systemic hyperuricemia through inhibition of uricase. Subsequently, the relevant assays were performed [31].

Apoptosis and intracellular ROS detection was performed according to the instructions of the manufacturers, followed by immediate flow cytometry (Beckman Coulter Life Sciences, Brea, CA, USA). All experiments were performed in triplicate, with data analyzed using FlowJo 10.8.1.

### 2.8. Western Blotting

Protein extraction from cell and mouse samples was performed using RIPA lysis buffer (Epizyme, Shanghai, China) supplemented with 1% protease and phosphatase inhibitors on ice for 30 min. The protein concentrations in the resulting supernatants were determined using a BCA kit (Beyotime, Shanghai, China). The prepared samples were then mixed with 5× loading buffer, denatured at 100 °C, and loaded onto 10% SDS-PAGE gels for separation. Proteins were then transferred to polyvinylidene fluoride membranes (Wuhan Servicebio Technology Co., Ltd., Wuhan, China), with nonspecific sites blocked by incubating with 10% skimmed milk powder for 2 h, and then the membranes were probed with primary antibodies (URAT1 (1:1000), GLUT9 (1:1000), ABCG2 (1:1000)) at 4 °C overnight. All antibodies came from Changsha Abiowell Biotechnology Co., Ltd. (Changsha, China). The membranes were then incubated with an HRP-conjugated goat anti-rabbit IgG secondary antibody for 2 h at room temperature, washed in Tris-buffered saline with Tween 20 (TBST), and Enhanced Chemiluminescence (ECL) solution was added to measure the optical density using ImageJ software (version 1.8, National Institutes of Health, Bethesda, MD, USA). β-actin was used as an internal reference.

### 2.9. Molecular Docking

The molecular structures of the primary active ingredients were retrieved from the PubChem database (https://pubchem.ncbi.nlm.nih.gov/) and saved in .sdf format. The three-dimensional crystal structures of bovine xanthine oxidase (XOD, PDB ID: 3NVW, 1.60 Å resolution, containing guanine as the co-crystallized ligand) and human URAT1 (PDB ID: 9JDV, 3.32 Å resolution, containing uric acid as the co-crystallized ligand) were obtained from the Protein Data Bank (PDB, http://www.rcsb.org/) in .pdb format. Water molecules and original ligands were removed from proteins using PyMOL Molecular Graphics System (version 2.4.0a0), with structures saved as PDB files. Docking pocket parameters were determined using the Getbox Plugin. Both processed protein and active component files were imported into AutoDock Tools 1.5.6 for conversion to PDBQT format. Molecular docking was then performed using AutoDock Vina 1.1.2. Finally, docking results were visualized and analyzed in PyMOL 2.6.0 and Discovery Studio 2019 [32].

### 2.10. Statistics

GraphPad Prism 8 (GraphPAD, San Diego, CA, USA) was used for statistical analysis. Data were analyzed using one-way analysis of variance. All results were expressed as the mean ± standard deviation (SD). No data points were excluded as outliers, and all valid experimental observations were included in the statistical analysis. *p* < 0.05 values indicated statistical significance.

## 3. Results

### 3.1. BrEE Chemical Composition Analysis

Preliminary UPLC-Q-TOF-MS/MS analysis, with reference to databases including Metlin, MassBank, and MoNA, tentatively identified 1072 compounds in BrEE. The total ion chromatogram (TIC) is shown (Figure S1). Fourteen compounds with peak areas exceeding 1 × 10^7^ were identified and are listed in Table 1, which collectively accounted for 75.37% of the total relative abundance in BrEE. Major compound classes included flavonoids, glucosinolates, and unsaturated fatty acids, and of these, quercetin-3-glucuronide was identified as the most abundant constituent, accounting for 22.04% of the total relative abundance, followed by gluconasturtiin (17.84%) and 4-methoxyglucobrassicin (13.39%).

### 3.2. BrEE’s Effects on Organ Indices and Serum Biochemical Parameters in HUA Model Mice

**Figure 2:**
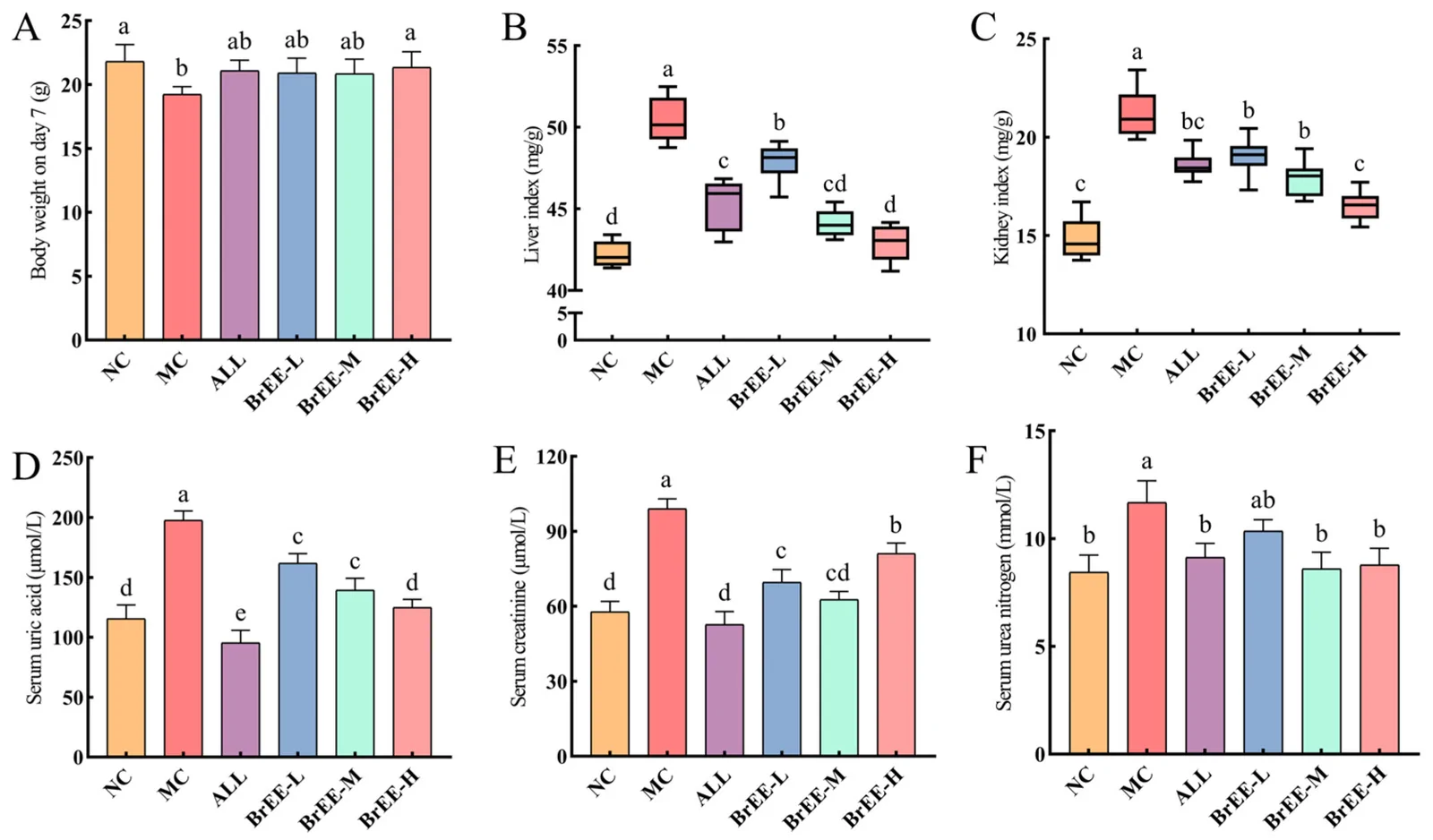
Mouse body weight (**A**), organ indices (**B**,**C**), serum uric acid levels (**D**), serum creatinine (**E**) and serum urea nitrogen concentrations (**F**). Data are expressed as the mean ± SD (n = 6). NC, normal control group; MC, hyperuricemia model group; ALL, allopurinol group; BrEE-L, low-dose BrEE group; BrEE-M, medium-dose BrEE group; and BrEE-H, high-dose BrEE group. Different lowercase letters indicate statistically significant differences among groups (*p* < 0.05), whereas groups sharing the same letter are not significantly different.

During the experimental period, all mice exhibited good mental status and smooth, glossy fur, with no significant abnormality in food or water consumption. MC (model control) mice exhibited slower body weight gain, and their body weights on day 7 were significantly different compared to NC mice (*p* < 0.05) (Figure 2A), while ALL and BrEE administration ameliorated this difference. Liver and kidney indices (Figure 2B,C) were significantly higher in MC mice compared to NC mice (*p* < 0.05), suggesting that PO and Hx potentially exerted renal and hepatic toxicity in animals. ALL and BrEE administration significantly reversed increased the organ indices in HUA mice (*p* < 0.05). Serum UA levels in MC mice were significantly elevated by 70.92% relative to NC animals (*p* < 0.05), indicating successful model induction. Following BrEE intervention, serum UA levels decreased in a dose-dependent manner. Specifically, in the BrEE-H group, serum UA decreased by 36.84% compared to the MC group and returned to levels comparable to NC mice (*p* < 0.05) (Figure 2D), while serum Cr and SUN contents decreased significantly (*p* < 0.05) (Figure 2E,F), indicating that BrEE has protective effects in the kidney.

### 3.3. XOD and ADA Activities Were Reduced by BrEE

**Figure 3:**
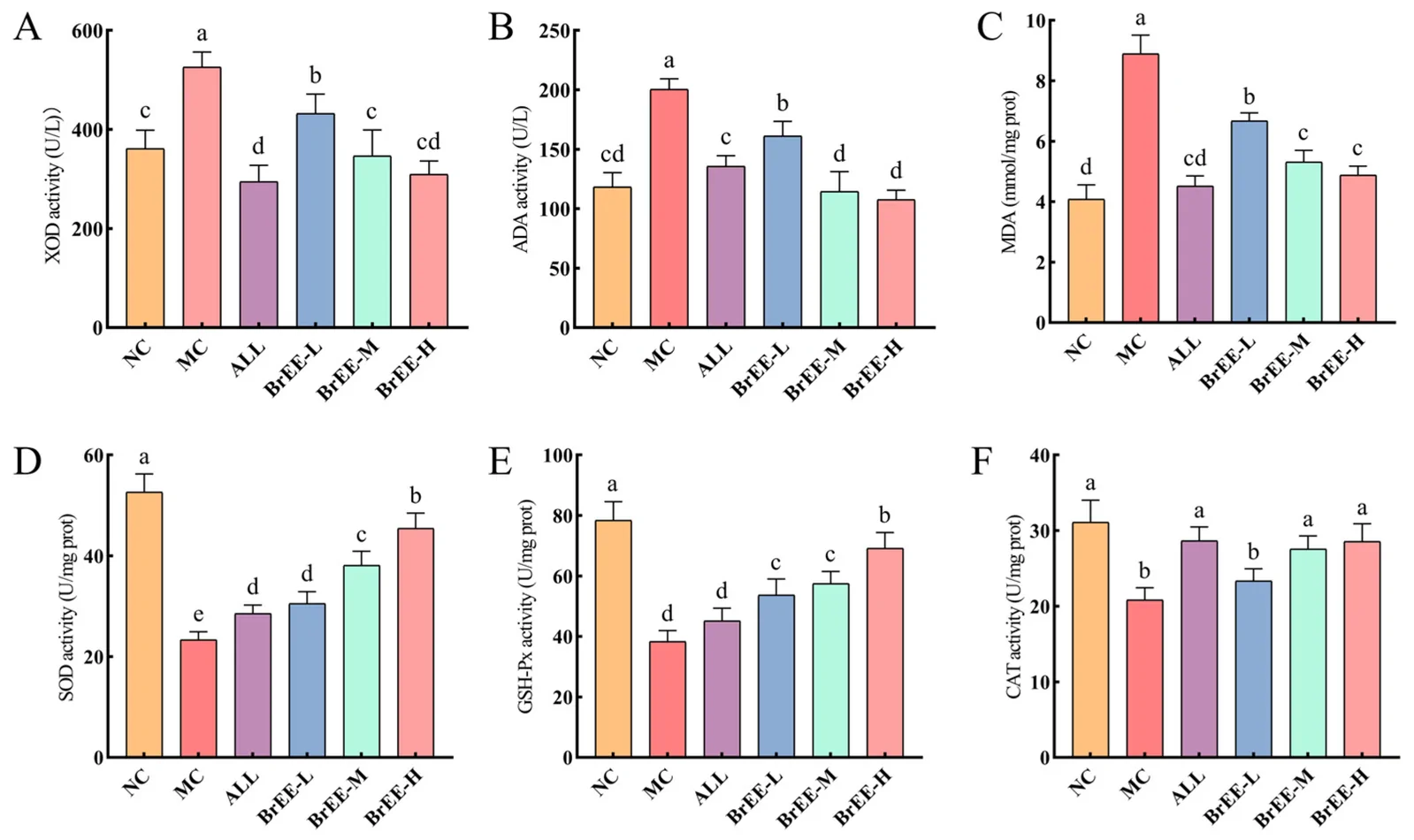
BrEE’s effects on activity of key purine-metabolizing enzymes involved in uric acid production (xanthine oxidase (XOD) and adenosine deaminase (ADA)) (**A**,**B**). BrEE’s effects on lipid peroxidation and antioxidant defense systems, as evidenced by MDA levels (**C**), and on the activity of the antioxidant enzymes SOD, GSH-Px, and CAT (**D**–**F**). Different lowercase letters indicate statistically significant differences among groups (*p* < 0.05), whereas groups sharing the same letter are not significantly different.

UA production is critically mediated by XOD and ADA. As shown in Figure 3A and Figure 3B, following Hx and PO modeling, compared with the NC group, hepatic XOD and ADA activities in MC mice increased by 45.50% and 69.54% (*p* < 0.05), respectively. ALL and BrEE administration effectively suppressed these elevated activities. In BrEE-H mice, ADA and XOD activities were 41.11% and 46.30% (*p* < 0.05) lower than those in MC mice, respectively, indicating that BrEE modulated purine metabolism-related enzyme activity, thereby reducing serum UA levels.

### 3.4. Oxidative Stress in HUA Mice Appeared to Be Ameliorated by BrEE

In kidney tissues from MC mice, MDA (lipid peroxidation end-product) levels were elevated compared to NC mice (*p* < 0.05) (Figure 3C). Conversely, the enzymatic activities of SOD, GSH-Px, and CAT exhibited a substantial decline (*p* < 0.05) (Figure 3D–F). Treatment with BrEE, especially in the medium- and high-dosage groups, successfully restored these pathological shifts, demonstrating a clear dose–response relationship. Specifically, administration of BrEE effectively reduced MDA formation and increased the activities of SOD, GSH-Px, and CAT, bringing these antioxidant parameters back to levels comparable to those of the NC group (*p* < 0.05). These findings suggest that BrEE provides robust protection against oxidative stress in HUA mice by both inhibiting lipid peroxidation and enhancing endogenous antioxidant enzyme activity.

### 3.5. BrEE Shows Protective Effects Against Renal Inflammation and Tissue Damage

**Figure 4:**
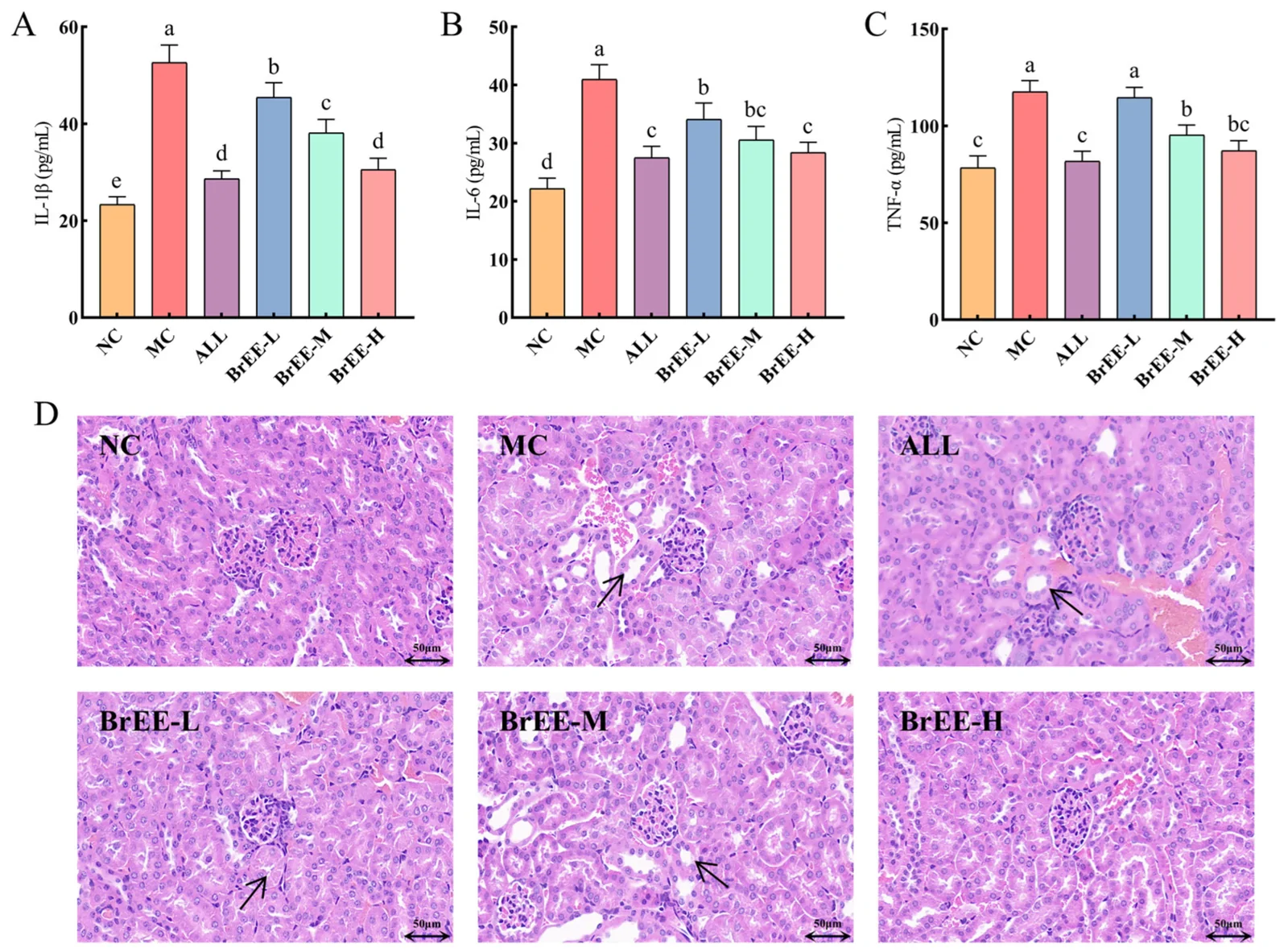
BrEE’s effects on renal inflammatory cytokine levels(IL-1β (**A**), IL-6 (**B**), and TNF-α (**C**)) in hyperuricemic mice. Representative renal histopathological sections from groups, as assessed by hematoxylin and eosin staining (Scale bar = 50 μm) Arrows indicate representative pathological alterations in the renal tissue (**D**). Different lowercase letters indicate statistically significant differences among groups (*p* < 0.05), whereas groups sharing the same letter are not significantly different.

Compared with the NC group, MC mice exhibited significantly up-regulated expression of renal pro-inflammatory factors, including IL-1β, IL-6, and TNF-α (*p* < 0.05). Medium- and high-dose BrEE significantly reduced these cytokine levels (*p* < 0.05) (Figure 4A–C), indicating anti-inflammatory effects in HUA-induced renal injury.

Representative photomicrographs of renal hematoxylin and eosin (H&E) staining are shown in Figure 4D. Kidney tissues from NC mice exhibited an intact architecture with clear glomerular contours, normal Bowman’s capsules, and well-arranged renal tubules. In contrast, MC mice displayed distinct pathological alterations, including glomerular atrophy, thickening of the wall of Bowman’s capsule, and tubular dilation, all of which are characteristic of HUA-induced nephropathy [33]. Notably, BrEE ameliorated these histopathological changes to varying degrees; the high-dose BrEE group demonstrated significant reno-protective effects, with renal architecture restored to near normal morphology.

### 3.6. BrEE Exerted Protective Effects Against UA-Induced Injury in HK-2 Cells

**Figure 5:**
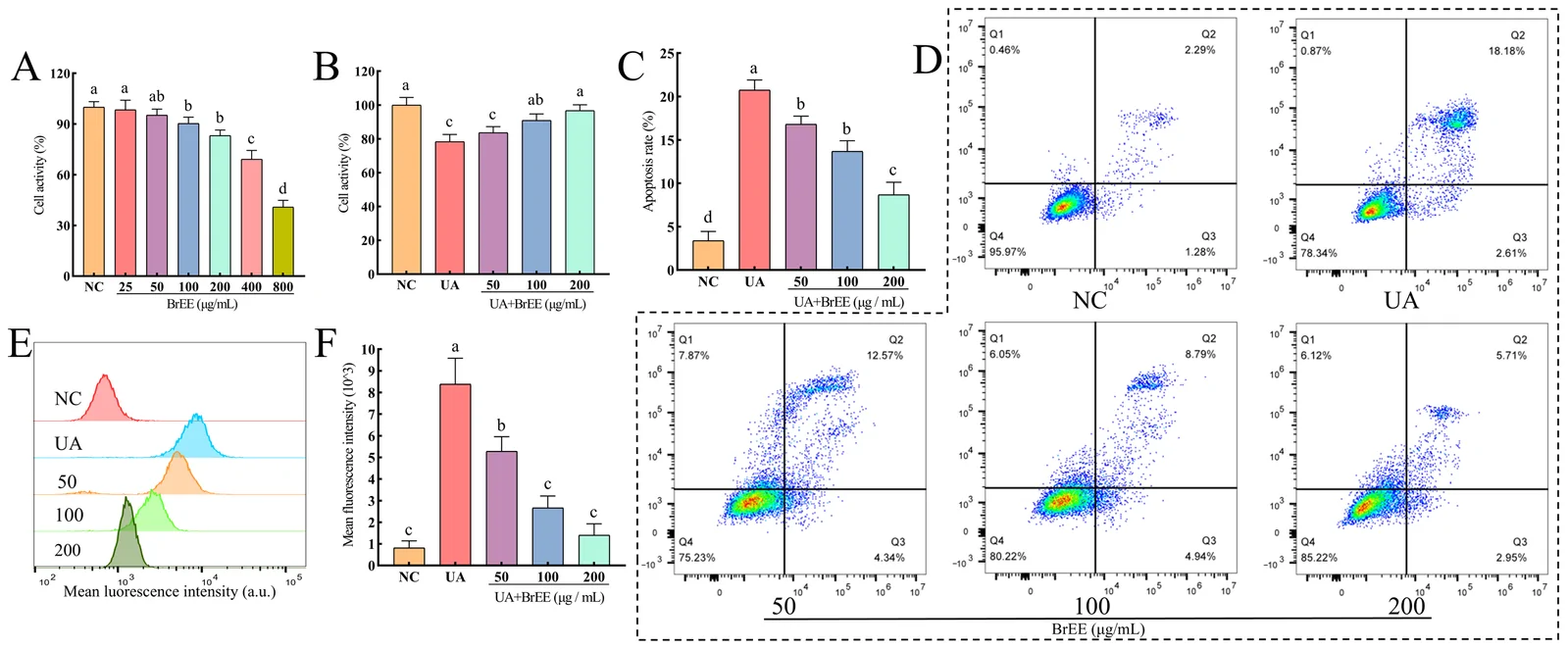
BrEE treatment effects at different concentrations for 24 h on HK-2 cell viability (**A**). Cell viability in different groups (**B**). Apoptosis status in different groups (**C**,**D**). BrEE reduced uric acid-induced reactive oxygen species production in HK-2 cells (**E**,**F**). Data are expressed as the mean ± SD from three independent experiments. Different lowercase letters indicate statistically significant differences among groups (*p* < 0.05), whereas groups sharing the same letter are not significantly different.

First, a safe BrEE concentration range in HK-2 cells was determined using CCK-8 assays. As shown in Figure 5A, treatment with BrEE in the 0–200 mg/L concentration range for 24 h did not significantly affect cell viability compared to untreated cells, indicating good biocompatibility of BrEE with cells in this concentration range. Therefore, 50, 100, and 200 mg/L concentrations were selected for subsequent experiments. Next, whether BrEE has protective effects against UA-induced injury in HK-2 cells was examined. Pretreatment with BrEE significantly increased cell viability compared with the UA group (Figure 5B). The total apoptosis in UA-treated cells was 20.75%, which represented a significant increase compared to control cells (3.38%) (*p* < 0.05). BrEE treatment also caused a dose-dependent reduction in apoptotic rates, with values decreasing to 8.68% in BrEE-H cells (Figure 5C,D), demonstrating effective BrEE mediated-protection from UA-induced apoptosis in HK-2 cells.

To investigate BrEE’s antioxidant effects at the cellular level, the fluorescent probe DCFH-DA was employed to evaluate intracellular ROS production. As shown in Figure 5E,F, UA stimulation significantly increased ROS levels in HK-2 cells compared to NC cells (*p* < 0.05). BrEE treatment effectively inhibited the UA-induced overproduction of ROS in a dose-dependent manner, indicating its significant capacity to alleviate oxidative stress in HK-2 cells.

### 3.7. BrEE Regulated the Protein Expression of URAT1, GLUT9, and ABCG2 in UA-Induced HK-2 Cells

**Figure 6:**
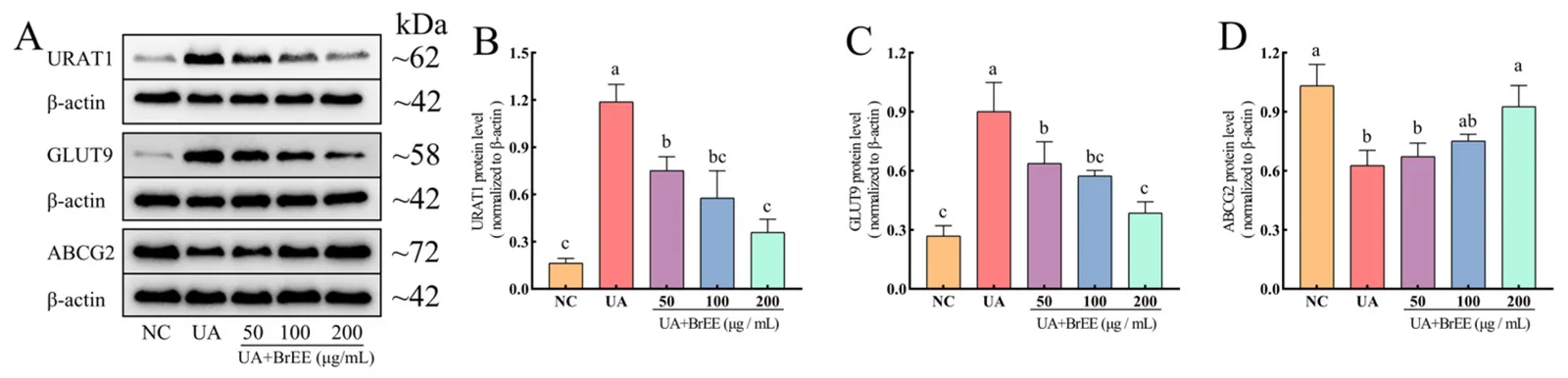
Representative Western blot bands of URAT1, GLUT9, ABCG2, and β-actin in HK-2 cells (**A**). Relative protein expression of URAT1 normalized to β-actin (**B**). Relative protein expression of GLUT9 normalized to β-actin (**C**). Relative protein expression of ABCG2 normalized to β-actin (**D**). Data are expressed as the mean ± SD from three independent experiments. Different lowercase letters indicate statistically significant differences among groups (*p* < 0.05), whereas groups sharing the same letter are not significantly different.

BrEE exhibited regulatory effects on the expression of multiple urate transporters in HK-2 cells. As shown in Figure 6, compared with the control group, UA stimulation significantly upregulated the expression of URAT1 and GLUT9 while downregulating the expression of ABCG2 in HK-2 cells. In contrast, BrEE treatment effectively reversed these alterations by suppressing UA-induced upregulation of URAT1 and GLUT9 while simultaneously enhancing ABCG2 expression. Notably, expression differences between BrEE-treated cells and UA model cells were statistically significant (*p* < 0.05).

### 3.8. Molecular Docking Results

**Figure 7:**
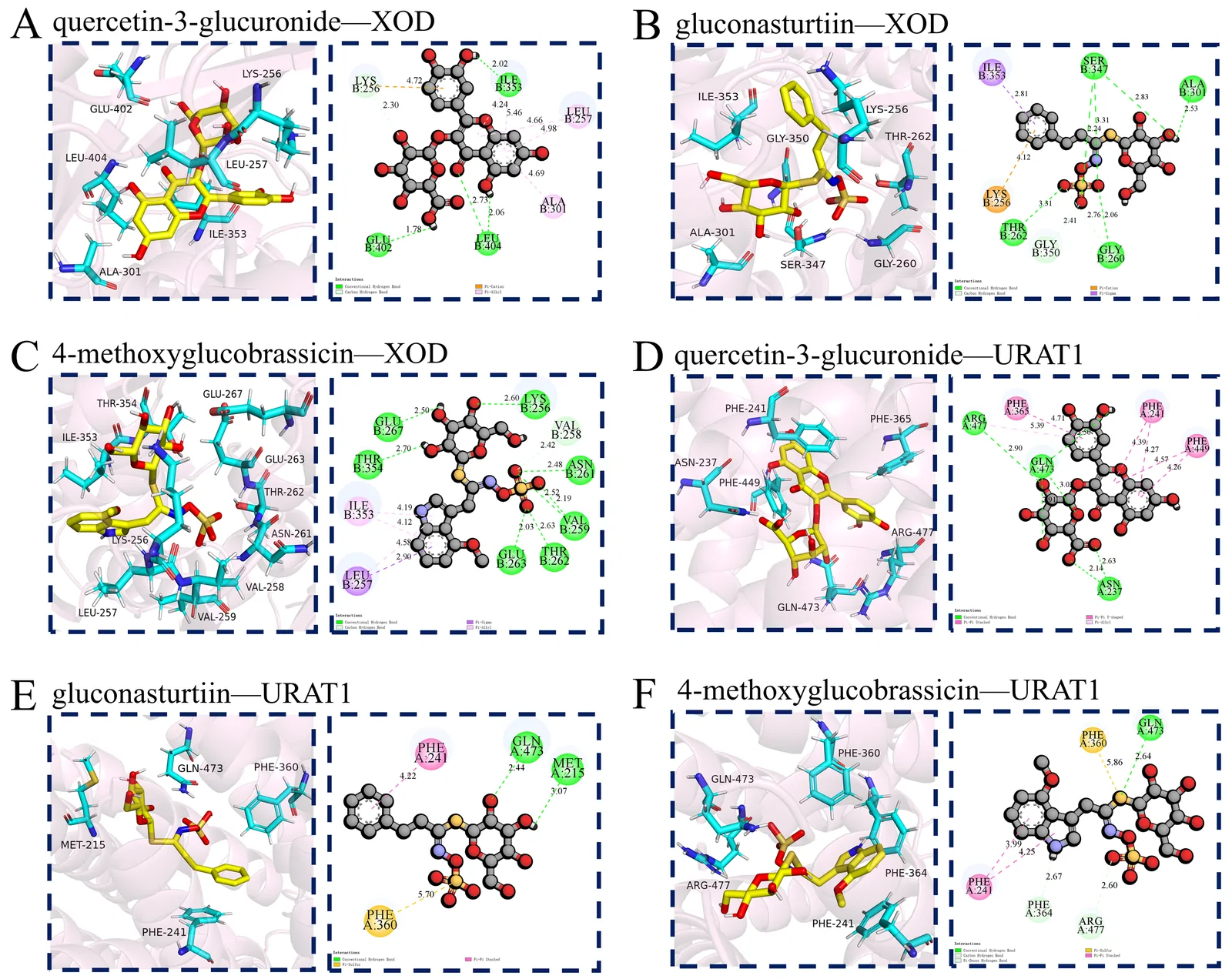
2D and 3D visualization of major BrEE compounds binding to XOD and URAT1 from molecular docking analysis. Quercetin-3-glucuronide–XOD (binding affinity = −9.8 kcal/mol) (**A**), gluconasturtiin–XOD (binding affinity = −9.0 kcal/mol) (**B**), 4-methoxyglucobrassicin–XOD (binding affinity = −9.0 kcal/mol) (**C**), quercetin-3-glucuronide–URAT1 (binding affinity = −8.4 kcal/mol) (**D**), gluconasturtiin–URAT1 (binding affinity = −8.3 kcal/mol) (**E**), 4-methoxyglucobrassicin–URAT1 (binding affinity = −8.3 kcal/mol) (**F**).

XOD and URAT1 are representative targets for uric acid production and renal urate reabsorption, respectively. The three most abundant compounds in BrEE, quercetin-3-glucuronide, gluconasturtiin, and 4-methoxyglucobrassicin, were selected for molecular docking with XOD and URAT1. Quercetin-3-glucuronide docked into the active site of XOD with a binding affinity of −9.8 kcal/mol, establishing four hydrogen bonds with residues ILE353, GLU402, and LEU404 (Figure 7A). Gluconasturtiin docked into the active site of XOD with a binding affinity of −9.0 kcal/mol, establishing six hydrogen bonds with residues GLY260, THR262, ALA301, SER347, and GLY350 (Figure 7B). 4-methoxyglucobrassicin docked into the active site of XOD with a binding affinity of −9.0 kcal/mol, establishing eight hydrogen bonds with residues LYS256, VAL259, ASN261, THR262, GLU263, GLU267, and THR354 (Figure 7C).

The binding energy between quercetin-3-glucuronide and URAT1 was −8.4 kcal/mol, with six hydrogen bonds formed, which involved ASN237, GLN473, and ARG477 (Figure 7D). The binding energy between gluconasturtiin and URAT1 was −8.3 kcal/mol, with two hydrogen bonds formed, which involved MET215 and GLN473 (Figure 7E). The binding energy between 4-methoxyglucobrassicin and URAT1 was −8.3 kcal/mol, with one hydrogen bond formed, which involved GLN473 (Figure 7F). In addition to hydrogen bonds, interactions between ligands and proteins involved pi–cation, pi–pi stacking, pi–alkyl interactions, and carbon–hydrogen bonds, all of which significantly increased their binding affinity.

It should also be noted that the favorable molecular docking results of the three major constituents of BrEE mentioned above with XOD and URAT1 do not demonstrate their direct involvement in the in vivo effects of BrEE. Molecular docking results should be regarded only as preliminary evidence of potential interactions. As the pharmacokinetic properties and in vivo exposure of these compounds were not determined in the present study, their actual contributions to the observed urate-lowering effects remain to be further elucidated.

## 4. Discussion

Inhibiting UA production by modulating purine metabolism enzymes represents a first line of treatment for HUA [34]. UA is the final purine metabolism product in humans and rodents, and XOD acts as the pivotal rate-limiting enzyme responsible for the sequential oxidation of Hx to xanthine and, ultimately, UA [12]. ADA also has important roles by converting adenosine to inosine, thereby providing substrates for downstream UA production [10]. In our study, hepatic XOD and ADA activities exhibited a prominent elevation in the HUA model mice; this enzymatic shift exacerbated purine degradation, leading to the overproduction of UA. A dose-dependent inhibition of XOD and ADA activities was observed following BrEE intervention (Figure 3A,B), leading to a substantial reduction in serum UA levels [35,36]. Consequently, BrEE likely acts as a potent modulator of the enzymatic machinery involved in purine metabolism, effectively curtailing UA biosynthesis from the outset. This mechanism was comparable to ALL, a first-line clinical drug for HUA treatment that lowers UA levels by inhibiting XOD activity [37]. Although BrEE is a complex mixture, its capacity to inhibit XOD and ADA activities indicates that its bioactive constituents may possess natural inhibitory activity against purine metabolism enzymes.

Previous studies have reported that flavonoids, including quercetin derivatives, bind to an active site in XOD and suppress its catalytic activity [38,39]. The high abundance of quercetin-3-glucuronide in BrEE suggests that such bioactive molecules are the primary drivers behind the extract’s suppressive effects on XOD and ADA activities. Compared with synthetic XOD inhibitors, natural products are often considered to have favorable biocompatibility. In the present study, the BrEE intervention effectively improved liver and kidney function-related indices, suggesting its potential as a promising approach for the long-term management of HUA. However, as the present study was not designed to systematically evaluate the toxicological profile of BrEE or to directly compare its safety with that of synthetic XOD inhibitors or other standard urate-lowering drugs, these findings should not be interpreted as evidence of superior biocompatibility or safety of BrEE relative to these agents. Further dose-dependent and long-term toxicity assessments, particularly evaluations of toxicity to the liver and other organs, are therefore required to comprehensively characterize the safety profile of BrEE.

The promotion of UA excretion through the modulation of renal urate transporter expression may represent another important mechanism contributing to the uric acid-lowering effects of BrEE. Renal uric acid handling involves processes such as filtration, reabsorption, secretion, and post-secretory reabsorption in the proximal tubules [40]. Among the transporters that regulate uric acid homeostasis, URAT1 and GLUT9 mediate the primary process of uric acid reabsorption. These proteins facilitate the vectorial transport of uric acid from the proximal tubular lumen back into systemic circulation [41,42]. In contrast, ABCG2 plays a crucial role in uric acid excretion by facilitating its transport into the urine and intestinal lumen [43]. In our UA-induced HK-2 cell model, UA stimulation significantly upregulated the expression of URAT1 and GLUT9 while downregulating the expression of ABCG2 (Figure 6), indicating a shift toward increased UA reabsorption and reduced excretion. These changes reveal the reasons for uric acid accumulation at the cellular level under hyperuricemia conditions. Notably, BrEE treatment effectively reversed these changes by suppressing URAT1 and GLUT9 expression and enhancing ABCG2 expression. These findings suggest that the urate-lowering effects of BrEE may be associated with alterations in the expression of key renal urate transporters, which may contribute to the restoration of the balance between urate reabsorption and excretion. However, as the functional urate transport activities of URAT1, GLUT9, and ABCG2 were not directly assessed, the observed changes in transporter expression should not be interpreted as direct evidence of corresponding alterations in transporter function. In addition, the effects of BrEE on the expression of URAT1 and GLUT9 were primarily investigated in UA-stimulated HK-2 cells. Although these findings provide preliminary evidence that BrEE may modulate the expression of renal urate transporters, their in vivo expression patterns and localization in renal tissue were not directly evaluated in the present study. Therefore, although the HK-2 cell model provides evidence for the potential involvement of renal urate transport-related proteins, the present findings cannot directly demonstrate that BrEE regulates these proteins in vivo. Accordingly, the observed changes should be interpreted as preliminary evidence supporting the potential involvement of renal urate transport in the effects of BrEE rather than as direct in vivo confirmation of this mechanism. Furthermore, the animal experiment in the present study was based on a short-term 7-day acute hyperuricemia model and was primarily designed to evaluate the urate-lowering and potential renoprotective effects of BrEE. In future studies, after further identification and isolation of the key bioactive compounds responsible for the observed effects of BrEE, it should be tested in longer-duration chronic hyperuricemia models. Complementary approaches, including immunohistochemistry and other protein-level analyses, could then be employed to systematically evaluate the in vivo expression, localization, and regulation of renal urate transport-related proteins. In addition, functional urate transport assays and targeted approaches, such as transporter inhibition or gene knockdown and overexpression, would further help clarify the functional and causal contribution of these transporters to the effects of BrEE.

Unlike benzbromarone, which is associated with hepatotoxicity and other adverse effects, BrEE demonstrated protective effects toward renal structures and function in our study. These findings highlight BrEE extracted from edible *B. rapa* L. (turnip) as a multi-target natural agent that can modulate urate transporters while simultaneously protecting renal cells from injury.

Renal protection is particularly important in HUA [44]. Excessive UA induces oxidative stress, mitochondrial dysfunction, and inflammatory responses in renal tubular cells, ultimately leading to structural damage and impaired renal function [40]. In our study, HUA mice exhibited significant renal oxidative stress, characterized by the accumulation of large amounts of MDA and an impaired antioxidant defense system, manifested as reduced antioxidant enzyme activity. These changes indicated excessive lipid peroxidation and a weakened endogenous antioxidant defense system under HUA conditions [45]. BrEE effectively alleviated oxidative stress by reducing MDA accumulation and restoring SOD, GSH-Px, and CAT activities in renal tissues (Figure 3). These findings suggest that BrEE reinforced the renal antioxidant defense architecture and thereby alleviated the redox imbalance provoked by uric acid overload. Similar to our in vivo experimental results, the HK-2 cell experiment revealed that BrEE treatment effectively weakened the surge in intracellular ROS levels triggered by uric acid exposure (Figure 5E,F). Given that ROS are a key upstream mediator of oxidative injury and inflammatory signaling, the antioxidant effect of BrEE may play a key role in its renal protective effects [46]. It should be noted that the present findings do not allow us to distinguish whether the antioxidant effects of BrEE are primarily directly or secondarily related to its urate-lowering activity. The observed decreases in MDA and improvements in SOD, GSH-Px, and CAT activities may result from the combined effects of a reduced urate burden and the intrinsic antioxidant potential of BrEE. Further studies using direct antioxidant assays and mechanistic approaches are required to distinguish the relative contributions of these pathways.

Persistent hyperuricemia promotes the synthesis and secretion of various inflammatory factors, notably IL-1β, IL-6, and TNF-α, within the renal microenvironment [47]. In our study, renal inflammatory cytokine levels were elevated in HUA model mice, indicating an enhanced inflammatory response. BrEE significantly suppressed the production of these inflammatory cytokines (Figure 4), demonstrating strong anti-inflammatory effects. Histopathologic analyses further supported these biochemical findings. H&E staining showed that HUA mice had the typical pathological changes, including glomerular atrophy, tubular dilation, and structural alterations, consistent with inflammation-mediated renal injury. However, the BrEE intervention ameliorated the renal histopathological alterations, particularly at higher doses, as evidenced by intact glomerular structures and normal tubular morphology in the treated groups. Thus, BrEE not only lowered serum UA levels, but also protected renal tissue integrity.

Previous studies have demonstrated that a variety of plant-derived extracts and natural products, including those from celery, cinnamon, and peony root, exert urate-lowering activities through mechanisms such as the inhibition of uric acid-producing enzymes and modulation of renal urate transporters [48]. Direct comparisons of efficacy across different studies are difficult because of substantial differences in plant sources, extraction procedures, chemical compositions, animal models, treatment durations, and dosing regimens. Therefore, the urate-lowering activity of BrEE should not be interpreted in isolation, nor should it be considered as evidence of superior efficacy or safety compared with other plant-derived agents. In this context, the value of the present study lies primarily in the integrated evaluation of BrEE across multiple aspects of urate homeostasis and HUA-associated renal injury.

In summary, elevated UA levels promoted ROS generation, which in turn activated inflammatory pathways and exacerbated renal injury, leading to further impaired UA excretion. By reducing UA production, enhancing its excretion, and suppressing oxidative and inflammatory damage, BrEE effectively alleviated the pathological symptoms of HUA. This multi-level protective mechanism may explain the superior overall efficacy of BrEE in ameliorating HUA.

## 5. Conclusions

Overall, BrEE exerted significant alleviating effects against HUA, which were associated with the regulation of purine-metabolizing enzymes and changes in the expression of renal urate transporters, together with antioxidant and anti-inflammatory effects. Both in vivo and in vitro experiments, along with molecular docking analysis, demonstrated that *B. rapa* L. is a promising source of bioactive compounds for the development of functional foods and dietary supplements aimed at regulating urate metabolism. Quercetin-3-glucuronide may represent a promising candidate for further investigation. Further studies are required to isolate the primary active monomeric compounds from BrEE, validate their specific anti-HUA efficacy, and elucidate their underlying molecular mechanisms. Additionally, long-term safety evaluations and clinical trials are necessary to fully assess the application potential of BrEE in HUA management.

## Figures and Tables

**Table 1:** The main components of BrEE identified using UPLC-Q-TOF-MS/MS.

No.	Name	Formula	m/z	Relative Abundance (%)
1	Quercetin-3-glucuronide	C_21_H_18_O_13_	477.07	22.04
2	Gluconasturtiin	C_15_H_21_NO_9_S_2_	422.06	17.84
3	4-Methoxyglucobrassicin	C_17_H_22_N_2_O_10_S_2_	477.07	13.39
4	(10*E*,15*Z*)-9,12,13-trihydroxyoctadeca-10, 15-dienoic acid	C_18_H_32_O_5_	327.22	4.68
5	(*Z*)-5,8,11-trihydroxyoctadec-9-enoic acid	C_18_H_34_O_5_	329.23	4.12
6	Glucoalyssin	C_13_H_25_NO_10_S_3_	450.06	3.01
7	Glucoerucin	C_12_H_23_NO_9_S_3_	420.05	1.94
8	Sulfo jasmonate	C_12_H_18_O_7_S	305.07	1.71
9	Myricetin 3-glucoside	C_21_H_20_O_13_	479.08	1.54
10	Gossypin	C_21_H_20_O_13_	479.08	1.43
11	Sinapoyl malate	C_15_H_16_O_9_	339.07	1.27
12	Myricetin-3-*O*- galactoside	C_21_H_20_O_13_	479.08	0.94
13	4-Methylpentyl glucosinolate	C_13_H_25_NO_9_S_2_	402.09	0.79
14	Glycerophospho-N- palmitoyl ethanolamine	C_21_H_44_NO_7_P	452.28	0.67

## Data Availability

The original contributions presented in this study are included in the article/Supplementary Material. Further inquiries can be directed to the corresponding authors.

## Supplementary Materials

The following supporting information can be downloaded at: https://www.mdpi.com/article/10.3390/foods15173108/s1, Figure S1: Total ion chromatogram of BrEE (TIC, negative ion mode).

## Abbreviations

The following abbreviations are used in this manuscript:

HUA	Hyperuricemia
UA	Uric acid
BrEE	*Brassica rapa* L. ethanol extract
HK-2	Human Kidney-2
XOD	Xanthine oxidase
ADA	Adenosine deaminase
URAT1	Urate transporter 1
GLUT9	Glucose transporter 9
ABCG2	ATP-binding cassette subfamily G member 2
UPLC-Q-TOF-MS/MS	Ultra-Performance Liquid Chromatography Quadrupole Time-of-Flight Tandem Mass Spectrometry
Hx	Hypoxanthine
PO	Potassium oxonate
SUN	Serum urea nitrogen
MDA	Malondialdehyde
SOD	Superoxide dismutase
GSH-Px	Glutathione peroxidase
CAT	Catalase
ALL	Allopurinol
ELISA	Enzyme-linked immunosorbent assay
IL	Interleukin
TNF	Tumor necrosis factor
CMC-Na	Sodium carboxymethyl cellulose
FBS	Fetal bovine serum
BCA	Bicinchoninic acid
ECL	Enhanced Chemiluminescence
TBST	Tris-buffered saline with Tween 20
TIC	Total ion chromatogram
H&E	Hematoxylin and eosin
CCK-8	Cell Counting Kit-8
