# Supplementary material for: Brassica rapa L. (Turnip) Ethanol Extract Alleviates Hyperuricemia by Modulating Purine Metabolism Enzymes and Renal Urate Transporters
Source: Foods. 2026 Sep 1;15(17):3108. doi: 10.3390/foods15173108 (PMC13564574; doi:10.3390/foods15173108)
Supplement: Supplementary file 1 [file foods-15-03108-s001.zip › foods-4493863-supplementary.pdf]

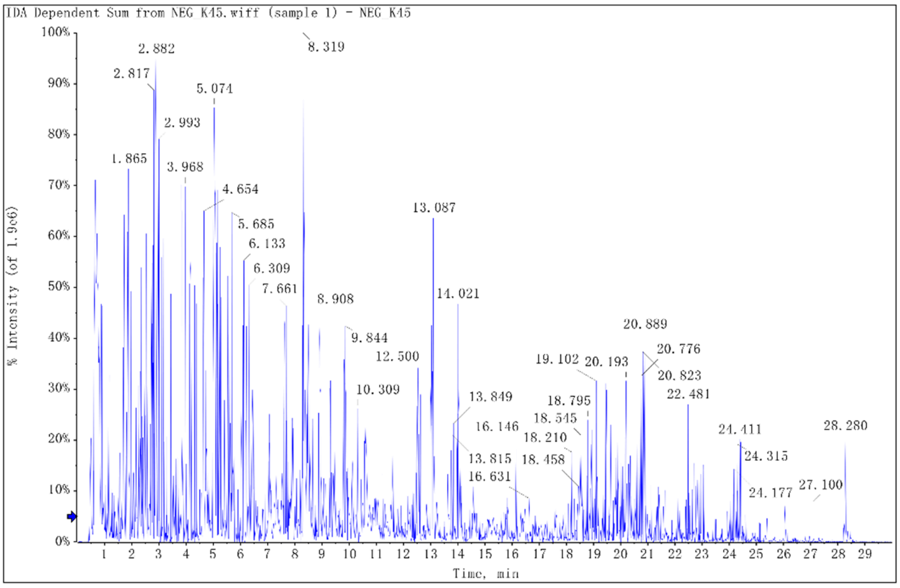

Figure S1. Total ion chromatogram of BrEE (TIC, negative ion mode).
